# Mandibular *Actinomyces *osteomyelitis complicating florid cemento-osseous dysplasia: case report

**DOI:** 10.1186/1472-6831-11-21

**Published:** 2011-07-21

**Authors:** Miller H Smith, Paul W Harms, Duane W Newton, Bill Lebar, Sean P Edwards, David M Aronoff

**Affiliations:** 1 Department of Oral and Maxillofacial Surgery, University of Michigan Medical School, 1150 W. Medical Center Drive, Ann Arbor, MI, 48109, USA; 2 Department of Pathology, University of Michigan Medical School, 1150 W. Medical Center Drive, Ann Arbor, MI, 48109, USA; 3 Clinical Microbiology Laboratories, Department of Pathology, University of Michigan Medical School, 1150 W. Medical Center Drive, Ann Arbor, MI, 48109, USA; 4 Division of Infectious Diseases, Department of Internal Medicine, University of Michigan Medical School, 1150 W. Medical Center Drive, Ann Arbor, MI, 48109, USA; 5 Department of Microbiology and Immunology, University of Michigan Medical School, 1150 W. Medical Center Drive, Ann Arbor, MI, 48109, USA

## Abstract

**Background:**

Apart from neoplastic processes, chronic disfiguring and destructive diseases of the mandible are uncommon.

**Case Presentation:**

We report, perhaps for the first time, the simultaneous occurrence of two such conditions in one patient, in a case that emphasizes the importance of bone biopsy in establishing the correct diagnosis. Florid cemento-osseous dysplasia (FCOD) is a chronic, disfiguring condition of the maxillofacial region. This relatively benign disease is primarily observed in middle-aged women of African ancestry. Cervicofacial actinomycosis is an uncommon and progressive infection caused by bacilli of the *Actinomyces *genus that typically involves intraoral soft tissues but may also involve bone. The accurate diagnosis of actinomycosis is critical for successful treatment. A diagnosis of osteomyelitis caused by *Actinomyces *bacteria was diagnosed by bone biopsy in a 53 year-old African-American woman with a longstanding history of FCOD after she presented with a new draining ulcer overlying the mandible.

**Conclusions:**

Clinicians should be aware of the possibility of actinomycosis arising in the setting of FCOD, and the importance of bone biopsy and cultures in arriving at a definitive and timely diagnosis.

## Background

Actinomycosis is a slowly-progressive infection caused by filamentous, gram-positive, anaerobic (or facultatively anaerobic) bacilli of the *Actinomyces *genus [1]. Such infections are characterized by suppurative and granulomatous inflammation with abscesses, tissue fibrosis, and the presence of draining sinus tracts or fistulae [2]. Actinomycosis usually spreads contiguously, ignoring tissue planes, and extruding bacteria-laden "sulfur granules" from erupting sinus tracts [3]. Cervicofacial infections are the most common manifestation of actinomycosis, although this is generally limited to the soft tissues without spreading to involve neighboring bone [1,2]. An odontogenic origin is typical for cervicofacial actinomycosis, which evolves as a chronic (or subacute) soft tissue swelling of the submandibular or paramandibular region [3].

The diagnosis of actinomycosis is easily missed because it mimics more common problems such as neoplasia [4]. In addition, actinomycetes are very sensitive to many common antimicrobials, so even a relatively few doses can render cultures negative [5]. However, it is important to make the diagnosis because actinomycosis can be disfiguring or even fatal, if vital structures (such as major arteries and airways) are involved [5]. And while antimicrobial therapy is effective against the actinomycetes, the treatment duration is remarkably long (6-12 months) [3]. Thus, the diagnostic and therapeutic challenges of actinomycosis underscore the need for an improved understanding of risk factors for infection and new information about clinical circumstances associated with this condition.

Florid cemento-osseous dysplasia (FCOD) is a disease with multiple bilateral and often symmetrically extensive lesions throughout the maxillofacial region (predominantly in the mandible) demonstrating a predilection for middle-aged African-American females [6,7]. Lesions are often asymptomatic and indolent in nature and may present as incidental findings on radiographs [6,8]. Occasionally patients present with dull pain or drainage from a prior extraction socket, or from a chronic irritation beneath a denture base, while some infrequently appear with expansion of the native bone [9,10]. Radiographically, the lesions present a mixed radiolucent and radiopaque "cotton wool" appearance with ill-defined borders with densely sclerotic irregularly shaped masses. Though they may occasionally look very similar in appearance to fibrous dysplasia, there is often a distinct sclerotic component with surrounding radiolucency [6,10,11].

Histopathologically, the lesions of FCOD demonstrate mature lamellar bone being replaced with thickened woven bone with curvilinear branching bony trabeculae, and separate masses closely resembling dental remnants such as cementum [7,9]. There is a high preponderance of fibrous tissue and osteoclasts but rarely inflammation unless secondary contamination results [7,9]. Chronic inflammation and infection may develop within the dysplastic densely mineralized tissue, which possesses a less robust blood supply, thereby resulting in sequestrum formation [6,8]. Given a preponderance of inflammation on pathology, some authors in the past have incorrectly characterized the lesions as diffuse sclerosing osteitis or chronic diffuse sclerosing osteomyelitis (CDSO) [9,12-17]. This characterization is incorrect as the multifocal bilateral lesions of FCOD represent a distinct diffuse pathologic spectrum despite occasional areas of inflammation, while CDSO lesions are unilateral and often contained within the mandible with universal inflammation [8].

Herein we report, possibly for the first time, the first association between these two chronic, disfiguring diseases, in a patient who presented with mandibular osteomyelitis caused by *Actinomyces *in the setting of underlying FCOD. We speculate that the infection was initiated when bacteria contaminated a denture-related ulceration of the soft tissues overlying the involved area. Clinicians should be aware of the pathogenesis of actinomycosis and the importance of bone biopsy and culture in making a timely diagnosis.

## Case presentation

A 53 year-old, edentulous, African-American woman with longstanding FCOD presented to her dentist with a 1 month history of swelling, pain, and purulent discharge involving the region of the left lower mandible. The drainage was described as white, thick, and malodorous. Radiographs revealed multifocal diffuse bony changes consistent with FCOD and a new radiolucency in the bone of the mandible underlying the swollen and draining soft tissues. She was treated with a chlorhexidine gluconate oral rinse and a two-week course of oral levofloxacin but did not improve. A biopsy performed approximately two months after the onset of symptoms revealed dead bone but no specific diagnosis. Two months after this biopsy the patient noted what appeared to be a bone fragment erupting from the same pus-draining ulcer. The ulceration and drainage continued and seven months after the onset of symptoms the patient was referred to our institution for further management. She denied fevers, chills, night sweats or other constitutional symptoms. Her dental history was significant for FCOD, a condition shared with her mother. The diagnosis of FCOD was established twenty five years prior to her presentation to the University of Michigan by biopsy coincident with the extraction of her remaining teeth. Her present dentures were more than 10 years old. Her medical history was significant for multiple sclerosis treated with interferon 1β and a venous thromboembolic disorder managed with oral warfarin.

**Figure 1:**
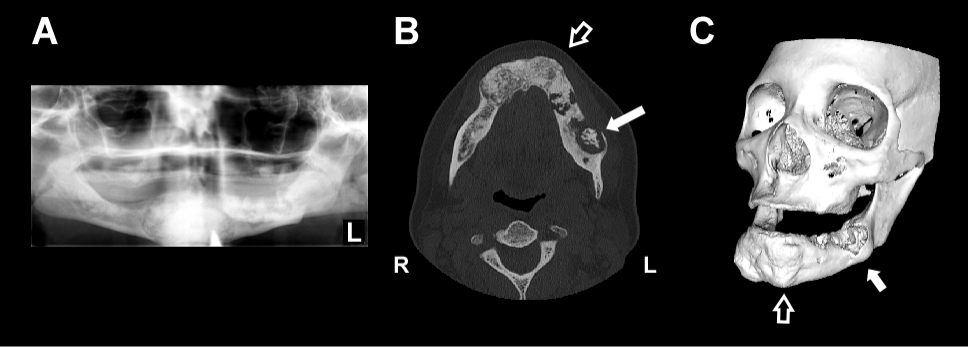
**Radiographic evidence of *Actinomyces *osteomyelitis complicating florid cemento-osseous dysplasia (FCOD)**. (**A**) Panoramic radiograph demonstrating mixed radiolucent and radiopaque lesions in the mandible with "cotton wool" appearance. Lesions are well demarcated with a radiolucent ring in all four quadrants though they are more subtle in the maxilla (**B**) Axial CT scan image showing hypertrophic, sclerotic and heterogeneous changes of FCOD within the mandible (open arrow). There is a large lytic lesion in the body of the left mandible with loss of bone at its lateral aspect and central sclerosis consistent with infection (solid arrow). (**C**) 3-dimensional CT image of generalized bony changes with expansion to maxilla and mandible consistent with FCOD (open arrow, corresponding to same location in panel A). There is focal erosion of left mandible in area of *Actinomyces *infection (solid arrow). CT images were reformatted with OsiriX imaging software (OsiriX Foundation).

On physical examination the patient's poorly-fitting mandibular denture was removed and the alveolar tissues revealed slight swelling along the anterior border of the ramus with an area of exposed bone measuring approximately 1 cm along the external oblique ridge laterally. There was soft tissue edema and congestion in this area. Her neck was otherwise supple without lymphadenopathy. She had no other oral lesions. Panoramic radiographs revealed bilateral lesions consistent with FCOD involving both the maxilla and mandible (Figure 1A). A maxillofacial computed tomography (CT) scan revealed midface, and mandibular findings consistent with FCOD (Figures 1B and 1C). On the patient's left, there was bony sequestrum within a radiolucent capsule to the anterior border of the ascending ramus (Figures 1B and 1C).

**Figure 2:**
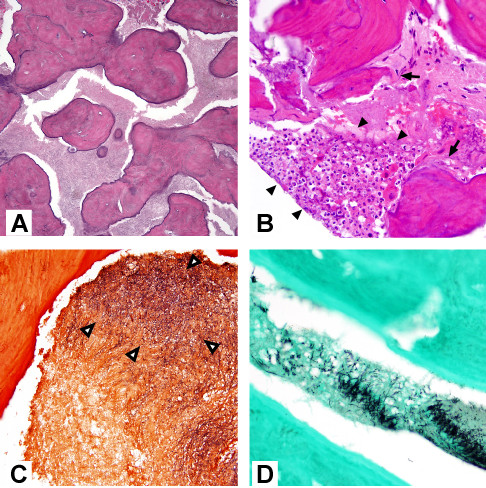
**Histopathological changes of *Actinomyces *osteomyelitis complicating florid cemento-osseous dysplasia (FCOD)**. (**A**) Excised mandibular bone revealed FCOD with irregular cementum droplets and rounded forms in a fibrovascular stroma (hematoxylin and eosin (H&E), magnification 200 ×). (**B**) Neutrophilic infiltrate (arrowheads) with adjacent necrotic bone (arrows) (H&E, magnification 400 ×). (**C**) Gram-positive filamentous organisms in marrow space (arrowheads) (Brown-Hopps stain). (**D**) Colonies of filamentous organisms in marrow (GMS).

Because of concern for chronic infection, the patient underwent debridement with bone biopsy and cultures of the diseased left mandibular ramus. The ulcerated soft tissues were repaired with a simple advancement flap. Purulence and necrotic bone were observed during this procedure. Histopathological examination of the biopsied bone revealed irregular trabeculae and bosselated cementum droplets in a fibrous stroma, typical of cemento-osseous dysplasia (Figure 2A). Changes consistent with osteomyelitis were also observed (Figure 2B). A Brown-Hopps tissue Gram stain revealed abundant gram-positive filamentous organisms consistent with *Actinomyces *(Figure 2C), which also stained positively with GMS silver stain (Figure 2D). Operative cultures yielded numerous (in quantity) *Actinomyces *species (not further identified to the species level) along with a mixture of oral anaerobic bacteria (table 1). Coagulase-negative *Staphylococcus *was the only aerobic bacterium isolated from the bone biopsy. Cultures for acid fast bacteria, fungi, and *Nocardia *were negative.

**Table 1:** Bacteria cultured from mandibular biopsy

Aerobic bacteria	Anaerobic bacteria
Coagulase-negative *Staphylococcus*	Numerous *Actinomyces spp*.
	*Lactobacillus spp*.
	α-hemolytic *Streptococcus*
	*Leptotrichia buccalis*
	*Capnocytophaga spp*.
	*Prevotella *spp.

The patient was initially treated for one week with oral amoxicillin combined with clavulinic acid 875 mg twice daily. However, because of the complicated polymicrobial nature of the infection, including bone involvement, therapy was changed to intravenous ertapenem one gram daily and the patient was treated for eight weeks. An additional 10 months of oral therapy were instituted with amoxicillin/clavulanic acid 875 mg twice daily. The patient responded well to antimicrobial treatment with complete healing of her operative site, and to date denies any further pain, swelling, or drainage.

This case newly demonstrates an association between two chronic, destructive and disfiguring conditions of the mandible, FCOD and *Actinomyces *osteomyelitis. It also emphasizes the utility of performing biopsies with appropriate aerobic and anaerobic cultures to establish a diagnosis in complicated cases such as this one. Initially our patient was felt to be suffering only from a progression of her FCOD coupled with ulceration caused by her poorly-fitting dentures. This led to a delay in her diagnosis. However, the eruption of purulent fluid from the ulcer and the eventual radiographic demonstration of an enlarged radiolucent ring surrounding more poorly-defined lesional tissue/sequestrum suggested that a superimposed osteomyelitis was present. While radiographs did not show a progressive osteolysis of the bone as might be expected typical osteomyelitis, the diagnosis was confirmed histopathologically. We speculate that our patient developed *Actinomyces *osteomyelitis following the mandibular ulceration.

Actinomycosis of the head and neck is an indolent infection that generally presents as a soft tissue swelling, mass, abscess, or ulceration of the oral-cervical region [1]. "Lumpy jaw" is caused when an actinomycete resident of the mouth (or other mucosal site) invades the underlying tissues through a loss of mucosal integrity [1]. The most common region for lesions to occur is the perimandibular area, but involvement of the bone is rare [1]. Anaerobic cultures are necessary to isolate the bacterium from pathological specimens, but culture-independent means of establishing the presence of these bacteria, such as PCR, are increasingly utilized [18]. Gram stains are more sensitive than cultures, perhaps because a lack of strict anaerobic processing or previous antibiotic use can render the cultures negative [1]. In cases of perimandibular infections, actinomycetes are often present in the setting of multiple other species of bacteria [1]. Although it is not always known how much the other bacteria are participating in the pathogenesis of infection, antimicrobial therapy is generally broad enough to cover these microbes. Although biopsy clearly demonstrated invasive actinomycosis, our patient had malodorous, purulent drainage from her mandible, suggesting that anaerobes or pyogenic bacteria were also present, which was confirmed by culture (table 1). It is likely that her infection was driven by this polymicrobial collection of pathogens and was not simply due to actinomycosis alone.

As evidenced in the present case, the treatment of cervicofacial actinomycosis requires attention to other identified pathogens, since these infections can be polymicrobial [3]. However, in many cases co-pathogens (such as oral anaerobic bacteria) are susceptible to the agents targeting the actinomycete. In cases where *Actinomyces *is the only identified pathogen then targeted therapy is warranted. Fortunately, actinomycetes remain exquisitely susceptible to beta lactam drugs and intravenous penicillin G remains a first-line agent [3]. For patients with intolerance to beta lactam agents, tetracycline agents can be substituted [2,3]. As detailed in a recent review [2], parenteral penicillin G, 10 to 20 million units daily divided every 6 hours for 4 to 6 weeks can be used for complicated cases, followed by oral penicillin V, 2 to 4 g/d divided four times daily for 6 to 12 months to prevent relapse [3]. The ultimate length of treatment depends upon clinical and pathologic response [2]. A number of alternatives to penicillin are available and include macrolides, tetracyclines, clindamycin, and carbapenem agents [2]. Importantly, many commonly used antimicrobials are not active against *Actinomyces *species and these include metronidazole, aminoglycosides, aztreonam, trimethoprim-sulfamethoxazole, penicillinase-resistant penicillins (e.g., nafcillin and oxacillin) and cephalexin [2,3]. In the present case ertapenem was used for the parenteral therapy because of the polymicrobial nature of the osteomyelitis and the convenience of once daily dosing.

It is interesting that our patient developed actinomycosis while on an immunomodulatory agent, interferon 1β, which was used to treat her multiple sclerosis. We were unable to identify previous reports of osteomyelitis associated with this medication. Infectious complications of interferon 1β are uncommon [19]. Although neutropenia can be a complication of therapy, our patient did not have any record of this while on therapy (data not shown).

FCOD is a benign lesion that can be disfiguring and lead to tooth loss, jaw fractures, and chronic infections. Although the etiology and pathogenesis of FCOD are unknown, the disease represents a disturbance in bone metabolism where normal bone becomes replaced by a connective tissue matrix that gradually develops cemento-osseous tissue [20]. As in our patient, the disease is more common in persons of African ancestry and can be familial [21]. FCOD is typically diagnosed through incidental findings on routine radiographic evaluation and biopsy often avoided to avoid the risk of chronically infected, non-healing wounds. The lesions are characteristic of a diffuse bilateral involvement of the maxillary and mandibular bones. Pathologic evaluation is often free of inflammation, however superinfection can occur and progress to osteomyelitis due to the limited blood supply of the dense bone [6,8,10]. Traumatic extractions and pressure ulceration caused by poorly fitting prosthetics can cause bone exposure which is susceptible to inflammation and infection of various organisms. Prior to the characterization of FCOD by Melrose in 1976 [9] these lesions were believed to represent CDSO [12-17]. Due to the dysplastic anatomy of the lesions, surgical intervention is often necessary for treatment of chronic infections combined with prolonged antibiotic therapy [8,10,11].

As observed in this case report, FCOD is characterized by fibrovascular stroma with a "ginger-root" pattern of irregular curvilinear trabeculae, as well as droplets of cementum ("cementicles") that fuse to form bosselated structures [22-24]. Coexisting bone cysts with fibrovascular lining may also be present [22,23], although these were not observed in the present case. Early-stage lesions of FCOD display fewer trabeculae and more prominent fibrovascular stroma, often with hemorrhage [22]. In the case presented here, these typical findings of FCOD were seen in conjunction with key features of *Actinomyces *osteomyelitis including filamentous gram-positive organisms, acute inflammation, and necrotic bone [25,26].

## Conclusions

In summary, we present a complicated association of two uncommon and destructive diseases of bone, FCOD and actinomycosis. The existing FCOD possibly contributed to a delay in establishing the diagnosis of actinomycosis because the deforming and destructive changes to the mandible produced by the infection were assumed to be due to progressing FCOD. Healthcare providers should be aware that actinomycosis can be an opportunistic pathogen of the mandible that can establish deforming and severe infections when a break in the integrity of the oral mucosa occurs. Proper cultures performed under anaerobic conditions are helpful and antimicrobial management should take into consideration the frequent polymicrobial nature of these infections.

## Abbreviations

FCOD: florid cement-osseous dysplasia.

## Informed consent

After approval by the Institutional Review Board of the University of Michigan Health System, written informed consent was obtained from the patient for publication of this case report and any accompanying images. A copy of the written consent is available for review by the Editor-in-Chief of this journal.

## Competing interests

The authors declare that they have no competing interests.

## Authors' contributions

All authors read and approved the final manuscript. MHS provided clinical care for the patient and wrote the manuscript. PWH provided expertise in the interpretation of tissue biopsies and assisted in writing the manuscript. DWN and BL conducted laboratory studies of the bacterial cultures isolated from the patient and provided expertise on the microbiology of *Actinomyces*. SPE provided clinical care for the patient and contributed to writing the manuscript. DMA provided clinical care for the patient, coordinated all aspects of this report, and wrote the manuscript.

## Pre-publication history

The pre-publication history for this paper can be accessed here:

http://www.biomedcentral.com/1472-6831/11/21/prepub
